# Arrhythmias in Cardiac Sarcoidosis: Management and Prognostic Implications

**DOI:** 10.3390/jcm13113165

**Published:** 2024-05-28

**Authors:** Suganya Arunachalam Karikalan, Ali Yusuf, Hicham El Masry

**Affiliations:** 1 Department of Cardiovascular Medicine, Mayo Clinic, Phoenix, AZ 85054, USA; arunachalamkarikalan.suganya@mayo.edu; 2 Department of Internal Medicine, Texas Tech University Health Sciences, Amarillo, TX 79430, USA; ali.yusuf@ttuhsc.edu

**Keywords:** cardiac sarcoid, ventricular arrhythmia, sudden cardiac death, arrhythmia, atrial fibrillation, ICD

## Abstract

Cardiac sarcoidosis (CS) is characterized by various arrhythmic manifestations ranging from catastrophic sudden cardiac death secondary to ventricular arrhythmia, severe conduction disease, sinus node dysfunction, and atrial fibrillation. The management of CS is complex and includes not only addressing the arrhythmia but also controlling the myocardial inflammation resultant from the autoimmune reaction. Arrhythmic manifestations of CS carry significant prognostic implications and invariably affect long-term survival in these patients. In this review, we focus on management of arrhythmic manifestation of cardiac sarcoidosis as well as risk stratification for sudden cardiac death in these patients.

## 1. Introduction

Cardiac sarcoidosis is a granulomatous inflammatory disease that occurs in patients with genetic predisposition. Cardiac involvement in sarcoidosis remains the most important contributor to mortality and is clinically manifest in at least 5% of patients with systemic sarcoidosis [1,2]. Although cardiac sarcoidosis (CS) remains a challenging diagnosis, the diagnostic rate of CS saw a 20-fold increase in the past 25 years, owing to the progress in cardiac imaging and increase in clinical suspicion [3,4,5].

The clinical manifestations of cardiac sarcoidosis are varied and include congestive heart failure and brady- and tachyarrhythmias, with sudden cardiac death as the presenting symptom in a substantial number of these patients [6,7]. Immunosuppression remains the mainstay of therapy in patients manifesting active inflammation secondary to CS. In addition, there is medical therapy for congestive heart failure as well as defibrillator implantation for patients who have a history or are at risk of sudden cardiac death [8,9,10].

In this narrative review, we focus on the current diagnostic, management, and risk stratification strategies for arrhythmias in CS patients.

## 2. Pathogenesis and Epidemiology

Sarcoidosis is a systemic disease characterized by the formation of infectious, non-caseating granulomas most commonly involving the lungs. The disease is believed to involve an inflammatory reaction involving both the innate and adaptive immune cells to an unknown antigen [11,12]. Several antigens have been suggested, including self-antigens (e.g., major histocompatibility complex), environmental and infectious particles (e.g., metals and inorganic substances, mycobacterium tuberculosis). Circulating monocytes interact with the antigen then differentiate into antigen presenting cells. This is followed by an aberrant reaction characterized by activation of CD4+ T cells expressing T-helper (Th-1, Th17 and Th17.1) and production of pro-inflammatory cytokines while the regulatory T-cell response (anti-inflammatory action) is impaired. This results in granuloma formation culminating in fibrosis and scarring secondary to cellular repair [13]. A genetic predisposition has been demonstrated in multiple studies after noting familial aggregation and ethnic variation of the disease. No clear mechanistic studies have been able to link this susceptibility to the pathogenesis of the disease [11].

The prevalence of cardiac sarcoidosis is not accurately known (many are clinically silent) but it is known that systemic sarcoidosis has a variable prevalence (8 to 11 per 100,000 in the US, 6.8 per 100,000 in Canada) depending on geographic and ethnic background. A higher prevalence is reported in Black Americans (141.4 vs. 49.8 100,000 Caucasians) in the United States and in Northern European countries such as Sweden and Canada (140 to 160 per 100,000) [14,15]. The average age at diagnosis is around 50 years with predominance of males in those diagnosed less than 50 versus higher incidence in females at older age [1,14,15,16].

### 2.1. Clinical Manifestations

The clinical presentation of CS is dependent on the extent and phase of the disease; CS can be classified as clinically silent when ECG abnormalities or imaging evidence of myocardial inflammation or scar are present in a patient with systemic sarcoidosis without cardiac-related symptoms. These patients would benefit from long-term follow-up and periodic re-evaluation of their cardiac status. Isolated CS is diagnosed when the heart is the only organ involved and has been associated with worse outcomes; the prevalence of isolated CS varies across the reported literature (26–75%) [11,17,18,19].

CS clinical manifestations are related to myocardial dysfunction or arrhythmias. Myocardial dysfunction results in congestive heart failure ranging from insidious symptoms to cardiogenic shock, especially in patients with extensive and severe myocarditis [1]. Arrhythmic manifestations of CS are variable and depend on the extent of myocardial involvement as well as the stage of the disease [1,20]. During the inflammatory phase, arrhythmias can result from direct involvement of the conduction system by granulomas (e.g., heart block) or myocardial inflammation resulting in proarrhythmia (e.g., Purkinje-mediated VT/VF or atrial fibrillation) and typically respond to immunosuppressive therapy. In contradistinction, the fibrotic phase of the disease promotes development of chronic scarring and hence reentrant arrhythmias (monomorphic VT) or permanent conduction block [1,20].

### 2.2. Diagnosis of Cardiac Sarcoidosis

The diagnosis of CS remains challenging and is typically one of exclusion. The suspicion for CS should arise in any unexplainable cardiac presentation, such as high-degree AV block, ventricular arrhythmias, sudden cardiac arrest, or heart failure. In patients with previously diagnosed extracardiac sarcoid, rise in heart failure markers such as troponin or Brain Natriuretic Peptide, and new-onset arrhythmias or heart failure should direct doctors towards CS diagnostic evaluation.

The presence of non-caseating granuloma on endomyocardial biopsy (EMB) remains the gold standard for the diagnosis of CS. Yet the patchy distribution, varied healing stages, and the midmyocardial to epicardial distribution of the granuloma result in a lower-yield EMB (20%). EMB guided by electroanatomic mapping to sample low-voltage areas has been used with better success. In this approach, intracardiac mapping is performed to define electrically abnormal tissue, then a bioptome is guided to the same area through a long sheath, doubling the yield of biopsy (41%) [21,22,23].

Diagnostic criteria for cardiac sarcoidosis have been proposed by the heart rhythm society (HRS) in 2014 and by the Japanese Circulation Society (JCS) in 2017 and are summarized in Table 1 [8,24,25]. The difference between the two proposed criteria is the emphasis on histological evidence of the disease in the HRS consensus, while it is only one of the diagnostic pathways in the JCS criteria. The HRS expert consensus allows for CS diagnosis in cases where the EMB is positive for non-caseating granulomas and other causes are excluded. If the EMB is nondiagnostic or not available, “probable CS” is suspected when extracardiac involvement is documented histologically along with specific cardiac findings. In general, ‘probable involvement’ is considered adequate to establish a clinical diagnosis of CS. The clinical pathway proposed by JCS includes major and minor criteria that encompass typical clinical presentation (high-grade AV block or VT) and imaging evidence consistent with CS. It has been our approach to pursue multimodality imaging in patients with suspected CS including echocardiogram, cardiac MRI, and PET-CT. The findings on echocardiogram are nonspecific and include regional wall motion abnormalities, LV aneurysm, and abnormal LV or RV strain. Recent studies suggest that abnormal LV and RV global strain are good predictors of CS.

Cardiac magnetic resonance imaging is an essential part of the initial work up of patients with suspected CS and allows for structural and functional assessment of both ventricles as well as assessment for the presence of fibrotic changes detected on late gadolinium imaging. CMR has a high sensitivity (90%) and specificity (77–85%) for the diagnosis of CS [26]. Scar burden quantified by LGE has been correlated with the risk of ventricular arrhythmias and the risk of sudden death [26].

PET imaging uses myocardial glucose metabolism to detect active inflammation; 18F-FDG PET is used to aid in the diagnostic work up of suspected cases when cardiac MRI is non-diagnostic and clinical suspicion remains. The sensitivity and sensitivity of FDG-PET ranges between 81–89% and 78–83%, respectively [27]. Importantly, we use this imaging modality in monitoring the disease activity and titration of the immunosuppression regimen until no significant uptake is demonstrated. Though FDG-PET detects inflammation in the myocardium, enabling detection of active disease and response to therapy, its value as a prognostic tool has not been established [17,28,29].

In patients with suspected CS, in addition to baseline ECG and echocardiogram, we typically obtain a cardiac MRI to assess for myocardial scar and edema. We then pursue FDG-PET imaging if the suspicion for CS remains. It is important to remember that in patients without a known history of sarcoid, chest imaging (CT) would be important to evaluate for pulmonary involvement and pursue bronchoscopy. The diagnostic yield for bronchoscopy and bronchoalveolar lavage along with lung/lymph node biopsy has been shown to be up to 84% in a study by Petek et al. [30]. Although EMB offers the opportunity to make a definitive diagnosis, its use is limited to a small proportion of those patients where the above work up is non-diagnostic [21,22,23,31,32]. 

## 3. Arrhythmic Manifestations of CS

### 3.1. Conduction Abnormalities

High-degree AV block is the most common conduction abnormality in patients with CS, arising due to infiltration/compression of the conduction system or coronary supply by granulomas or due to scar tissue formation [33]. Reversibility of the conduction block after initiation of steroid therapy is correlated to resolution of the inflammation and is seen in up to 43% of patients [34]. Importantly, CS is an important etiology of idiopathic Mobitz II and third degree block; in a population of young and middle-aged adults with high-degree conduction block, one-third of the cohort (10/30) had a diagnosis of CS, as reported by Maizel et al. [33]. Presentation with HF symptoms, thick IVS, and RV dysfunction on echocardiography was significantly associated with the CS cohort [33,35]. Similarly, in a subanalysis of the ILLUMINATE-CS registry, AV block was observed in 37–51% of the 511 patients with higher incidence in the older age group (the increased incidence in older patients can be rationally attributed to the degenerative age-related AV block). In view of the above data, screening for the disease in patients <60 years old who present with unexplained Mobitz type II or third-degree AV block has been recommended by the HRS 2014 consensus (class 2a) [6,8].

Additional electrocardiographic manifestations of conduction system disease like first-degree AV block, wide QRS with typical bundle branch block, or IVCD are common in patients with CS with a variable prevalence across the reported literature (up to 61%) with RBBB being more common. These abnormalities are non-specific and are clinically useful as a possible marker of cardiac involvement in patients with systemic or pulmonary sarcoidosis, prompting further screening. 

### 3.2. Atrial Arrhythmias and Sinus Node Dysfunction

Atrial lesions are found in only 6.7% to 11% of patients [36,37]. The pathophysiology of atrial fibrillation in CS is multifactorial; it could be related to direct granulomatous involvement of the atria but also to remodelling related to ventricular dysfunction, elevated filling pressure, or pulmonary hypertension (secondary to lung involvement). The incidence of AF was variable across the reported literature; in the post hoc analysis study of the ILLUMINATE-CS registry, 10% of patients had atrial fibrillation at the time of CS diagnosis, and it was significantly associated with increased HF hospitalization and cardiac death [38]. In the study by Niemala et al., in a cohort of 118 AF-naïve CS patients, 29% developed atrial arrhythmia over a median follow-up of 3 years, of which AF was persistent in 21% and permanent in 12%. The cumulative incidence of developing AF at 5 years from diagnosis was 30% [39]. Atrial 18F-FDG uptake on PET scans along with left atrial enlargement detected on echocardiographic and CMR evaluations was a strong independent risk factor for AF development. The role of FDG-PET in AF risk prediction was evaluated by Niemala et al., where atrial FDG uptake was an independent and strong predictor of AF events (HR: ~6.0) [39]. The estimated 5-year incidence of AF was 55% in patients with positive atrial FDG activity compared to only 18% in CS patients without atrial FDG uptake. Despite being a less lethal condition than VAs, patients are frequently symptomatic, requiring medical therapy for rate and rhythm control as well as anticoagulation. The stroke risk has not been studied specifically in patients with CS but using the CHADS-Vasc score is recommended, and importantly, screening those patients for AF is recommended, especially those that demonstrate positive atrial FDG uptake on PET scanning [19,40,41].

Sinus node dysfunction has been reported in CS and can manifest similarly to conduction abnormalities. Whether this is related to direct involvement of the atrial tissue around the sinus node or ischemia/infarction of the coronary blood supply is not definitive. Similarly, the exact incidence of sinus node dysfunction in these patients is not well known [42,43,44].

*Ventricular arrhythmia:* Sustained ventricular tachycardia and aborted sudden death is the second most common clinical presentation of manifest CS (30%) [45]. More importantly, VAs are the most important mode of death in CS, irrespective of the initial presentation; hence, risk stratification of sudden cardiac death is a cornerstone in the management of those patients. The mechanism of arrhythmia is believed to be most commonly reentry related to scar formation and is consistent with the increased incidence of VT in those patients manifesting a significant burden of LGE on CMR. Additional mechanisms could be at play, including (1) Purkinje-mediated factors, which may explain some of the cases of VT/VF storm in patients with extensive inflammation, and (2) bundle branch reentry in those with significant conduction disease and widened QRS. Several studies evaluated predictors for recurrent arrhythmias and included clinical, electrocardiographic, and imaging characteristics in CS patients. EF is an important but not the sole predictor of VA and a multifactorial approach to risk stratification is recommended and is described below. Retrospective studies evaluating CS patients presenting with AV block were remarkable for a significant (24%) 5-year incidence of SCD and VT in those with preserved or mildly reduced EF [46]. 

In a nationwide Japanese cohort study, ventricular arrhythmic events were significantly higher in patients with concomitant NSVT and AV block, and concomitant NSVT and abnormal 67Ga scintigraphy or 18F-FDG PET of the heart at CS diagnosis than in patients without the concomitance [47]. Male sex, syncope, lower EF, ventricular pacing, complete AV block, RV dysfunction, and symptomatic heart failure were each predictors of VA and ICD shocks in patients with CS [48,49,50,51,52]. 

The presence of LGE on CMR remains the most important predictor of ventricular arrhythmias in patients with CS. In a cohort of 205 patients followed for a mean of 36 ± 18 months, Murtagh et al. demonstrated that the risk of death and VT was more than 20× higher in patients with compared to those without LGE in a cohort of 205 patients [53,54]. For every 1% increase in LGE burden, the hazard of death/VT increased by 8% despite a preserved EF. Right ventricular involvement is particularly pro-arrhythmic, and RV LGE was found to have a 100% positive predictive value for adverse events, including VA and death, in the multicenter registry for cardiac sarcoidosis [55]. It is not surprising that the extent of scar formation is related to arrhythmogenesis and a cutoff value of >6% LGE burden is accepted as the predictive threshold for adverse events. In the prospective study by Smedema et al., who followed 84 patients with biopsy-proven pulmonary sarcoidosis for a median of 56 months after baseline CMR [56,57], many of these patients (70%) were asymptomatic from a cardiac perspective and only a small proportion had LV systolic dysfunction (19% with EF of <50%). Biventricular LGE was the strongest independent predictor of the composite endpoints of congestive heart failure hospitalization, sustained ventricular tachycardia, appropriate ICD therapy, pacemaker implantation for high-degree atrio-ventricular block, or cardiac death (multivariate Cox regression HR 10.2 and *P* < 0.001). The 7% LV LGE burden was derived by ROC curve analysis and demonstrated modest ability to predict composite outcomes with 35% positive and 95% negative predictive values. Another retrospective cohort study of 290 CS with ICD found that refinement of the LGE threshold to 5.7% was sensitive and specific in predicting VA and SCD outcomes [58].

### 3.3. Treatment

**Figure 1:**
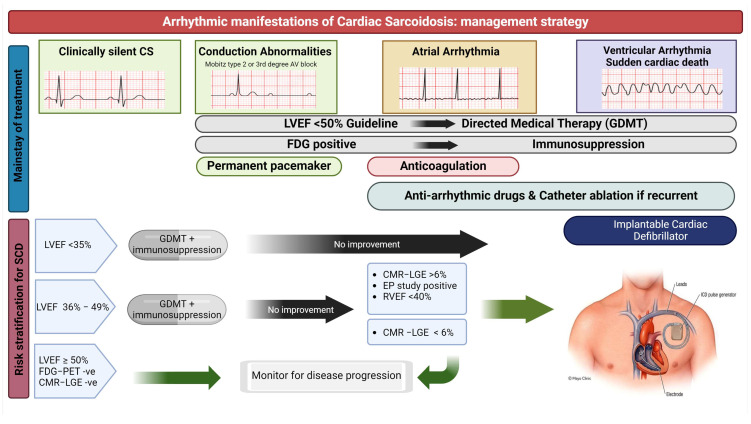
Central Illustration.

Immunosuppression with corticosteroids has been the mainstay in the treatment of symptomatic CS and is guided by the presence of inflammation on cardiac imaging or EMB (Figure 1). With the lack of randomized clinical trial data, questions remain regarding the duration of treatment and response of different arrhythmias to the immunosuppression [34,59,60,61]. Shorter duration of use can result in recurrence of the disease and longer duration of use exposes the patient to the risk of side effects and impaired quality of life [62]. Steroid-sparing agents like methotrexate, mycophenolate mofetil, and azathioprine as second-line treatment and TNF inhibitors such as infliximab, rituximab, and adalimumab as third-line drugs serve as a maintenance regimen [62,63]. The response to therapy is monitored through improvement of symptoms, levels of inflammatory markers such as BNP, soluble interleukin 2 receptor (sIL-2R), and serum adenosine deaminase 2 (ADA-2) levels, ECG and echocardiographic worsening or recovery [64]. CMR has limited ability in assisting with monitoring due to the interference of intracardiac devices [65,66]. FDG-PET is a preferred modality for monitoring treatment response and surveillance for any recurrence [63,67]. 

Although a significant recovery of systolic function is expected with immunosuppressive therapy in patients demonstrating active inflammation and low or no burden of scarring on MRI imaging, it is difficult to predict the response of different arrhythmias [68]. AV block has been shown to resolve in many CS patients (42%) with steroid therapy [34]. Resolution of atrial arrhythmias in CS patients treated with steroid has been reported in small case series. Similarly, there is a paucity of data to guide immunosuppressive therapy in the management of VA; it is common to see an increase in the burden of PVCs and non-sustained VT in CS patients with positive FDG [34]. It is our practice to use high-dose steroid in the treatment of CS patients presenting with VT/VF storm and evidence of active inflammation. 

#### 3.3.1. Device Implantation and Risk Stratification for Sudden Cardiac Death

Sudden cardiac death and ventricular arrhythmia remain the most feared complications of CS and the most common cause of death in these patients [46,47]. Risk stratification for SCD remains challenging due to the lack of large prospective studies, EF being a suboptimal predictor of SCD, and the multitude of predictive factors suggested in the available literature (Figure 1). We summarize below the available evidence and society recommendations (HRS 2014 and ACC 2017) and our approach [8,58]:

1. In CS patients presenting with Mobitz-II or third-degree AV block, permanent pacing is indicated even when conduction normalizes with steroid therapy (due to risk of recurrence). In those patients, ICD implantation for primary prevention is reasonable (class IIa) due to increased risk of SCD and VA.
2. Survivors of sudden cardiac death or those presenting with sustained VA have a clear indication for implantation of an ICD for secondary prevention (class I).
3. Those with persistent severe LV systolic dysfunction (LVEF < 35%) despite optimal medical therapy and immunosuppression, if active inflammation is demonstrated, are candidates for ICD implantation for primary prevention (class I). Expert opinion suggests proceeding with ICD implantation without a waiting period in those with severely depressed EF of less than 25% due to low likelihood of EF recovery [69,70].
4. Patients with mild to moderate LV systolic dysfunction (35 < EF < 50%) and concomitant RV dysfunction with RV EF < 40%, despite optimal medical therapy and immunosuppression, if active inflammation is demonstrated, might be considered for ICD (class IIb). In these patients, we typically assess for high-risk features—namely an LGE burden ≥ 6% or history of unexplained syncope—that might consolidate the decision to proceed with an ICD (class IIa).
5. Stratification of patients with preserved or mildly reduced LV systolic function represents a significant challenge due to a relevant risk of sudden death. We use CMR as an important tool in the process; lack of LGE has a high negative predictive value for VA and no further work is needed. In those with significant LGE or biventricular LGE, an electrophysiologic study (EPS) can be considered; inducible sustained VT would be an indication for ICD implantation [71,72]. The role of EPS in CS patients was evaluated in two studies demonstrating a good negative predictive value [73,74].

The majority of CS patients implanted with an ICD receive a transvenous system that has the distinct advantage of anti-tachycardia pacing (ATP) to address some of their VT in comparison to a subcutaneous system. The relatively young age at implant and concomitant immunosuppression use puts these patients at increased risk of long-term complications. The advent of extravascular ICD might prove particularly useful in CS patients, as it provides the option of ATP along with a lower risk of complications.

#### 3.3.2. Anti-Arrhythmic Therapy and VT Ablation

In patients with recurrent ventricular arrhythmias, antiarrhythmic therapy, usually a class 3 agent along with beta-blockers, is used (Figure 1). Amiodarone is the most commonly used agent, especially in those patients with significant LV systolic dysfunction. It is important to consider the potential side effects of chronic amiodarone use in CS patients who commonly have pulmonary involvement. The value of a multidisciplinary approach cannot be overemphasized and close monitoring for new or worsening respiratory symptoms and pulmonary function is recommended. VT ablation in CS patients with refractory arrhythmia is recommended after ensuring adequate control of the active inflammation with immunosuppressive therapy. A significant proportion of patients will necessitate both endocardial and epicardial ablation and multiple circuits are typically encountered. Although significant reduction in ICD shock and risk of recurrent VT storm was demonstrated in recent studies, the outcomes of VT ablations in CS are relatively modest with a significant risk of recurrence (55%) and need for repeat ablation (37%) [75,76]. 

Cardiac sympathetic denervation (CSD): CSD has been used as a bailout strategy for the management of patients with refractory VA. Acutely, stellate ganglion block using an amide local anesthetic is used to rescue patients with VT/VF storm and recurrent ICD shocks. Stellate ganglion block results in a significant reduction in the noradrenergic input to the myocardium and hence the genesis of many ventricular arrhythmias. Long-term effects can be achieved using thoracoscopic bilateral surgical excision of the distal half of the stellate ganglion with an acceptable risk of complications [77]. Small case series have shown CSD to be successful in reducing the rate of VT recurrence and ICD shocks in CS patients with refractory VA [78]. 

Heart transplantation and Left ventricular assist device (LVAD): Patients with CS have shown good outcomes after heart transplant [35]. However, CS recurrence after transplant has been reported. In the meta-analysis by Buttar et al., with pooled data for 42 patients, the mean survival was 71.4 months with CS recurrent in 3/30 patients [79]. Fortunately, data from the UNOS 1990–2020 registry showed no significant differences in short- or long-term outcomes post-heart transplant in patients with CS and NICM. Recurrence of CS was observed in only 5% to 10% patients, especially after cessation of steroids [80]. A left ventricular assist device is typically used in patients with CS with refractory heart failure both in the acute setting (fulminant myocarditis and cardiogenic shock) or progressive pump failure [81]. Although not many reports regarding the outcomes after LVAD implant in CS are available, Rosenbaum et al. reported 1-year and 5-year survival rates of 82% and 55%, respectively [82]. Ventricular arrhythmias continue to be a risk post-LVAD implantation; hence, careful patient selection is recommended and continued antiarrhythmic therapy or even intraoperative ablation are reasonable considerations.

## 4. Conclusions

Cardiac sarcoidosis continues to pose a significant challenge to clinicians both from a diagnostic as well as management perspective. Cardiac arrhythmias are commonly seen during the initial presentation of the disease and sudden cardiac death remains the most feared complication and the primary cause of mortalities in these patients. CS therapy is typically multifaceted and requires a multidisciplinary team to coordinate immunosuppressive therapy, heart failure treatment, device implantation, as well as risk stratification for sudden cardiac death. Large-scale registries and prospective trials might offer additional insights into medical management of these patients as well as SCD prevention. 

## Figures and Tables

**Table 1:** Comparison of current diagnostic criteria for cardiac diagnosis.

	JCS 2016/2017	HRS Expert Consensus 2014
**Histological diagnosis**	EMB or surgical specimen with non-caseating epithelioid granulomas	EMB with non-caseating granuloma on histological examination of myocardial tissue with no alternative cause
**Clinical diagnosis**	Negative EMB or ineligible for EMB Epithelioid granulomas in organs other than heart + clinical cardiac involvement Clinical findings suggestive of pulmonary or ophthalmic sarcoidosis + 2/5 of characteristic laboratory findings of sarcoidosis	Histological extra-cardiac sarcoidosis +
**Criteria for cardiac involvement**	≥2/5 major criteria or 1/5 major criteria + ≥2/3 minor criteria Major: (a) High-grade atrioventricular block (including complete atrioventricular block) or fatal ventricular arrhythmia (e.g., sustained ventricular tachycardia and ventricular fibrillation) (b) Basal thinning of the ventricular septum or abnormal ventricular wall anatomy (ventricular aneurysm, thinning of the middle or upper ventricular septum, regional ventricular wall thickening) (c) Left ventricular contractile dysfunction (left ventricular ejection fraction less than 50%) or focal ventricular wall asynergy (d) 67Ga citrate scintigraphy or 18F-FDG PET reveals abnormally high tracer accumulation in the heart (e) Gadolinium-enhanced MRI reveals delayed contrast enhancement of the myocardium Minor: (f) Abnormal ECG findings: Ventricular arrhythmias (nonsustained ventricular tachycardia, multifocal or frequent premature ventricular contractions), bundle branch block, axis deviation, or abnormal Q waves (g) Perfusion defects on myocardial perfusion scintigraphy (SPECT) (h) Endomyocardial biopsy: Monocyte infiltration and moderate or severe myocardial interstitial fibrosi	One or more of Steroid +/− immunosuppressant-responsive cardiomyopathy or heart block Unexplained reduced LVEF (o40%) Unexplained sustained (spontaneous or induced) VT Mobitz type II 2nd degree heart block or 3rd degree heart block Patchy uptake on dedicated cardiac PET (in a pattern consistent with CS) Late Gadolinium Enhancement on CMR (in a pattern consistent with CS) Positive gallium uptake (in a pattern consistent with CS) + Other causes for the cardiac manifestation(s) have been reasonably excluded
**Isolated Cardiac Sarcoidosis**	Prerequisite (1) No clinical findings characteristic of sarcoidosis are observed in any organs other than heart. (2) 67Ga scintigraphy or 18F-FDG PET reveals no abnormal tracer accumulation in any organs other than the heart. (3) A chest CT scan reveals no shadow along the lymphatic tracts in the lungs or no hilar and mediastinal lymphadenopathy (minor > 10 mm). Histological: EMB/surgical specimens demonstrating non-caseating epithelioid granuloma Clinical: Criterion (d) + 3/5 major criteria	

## Abbreviations

AF, atrial fibrillation; ATP, anti-tachycardia pacing; AV, atrio-ventricular; BNP, brain natriuretic peptide; CMR, cardiac magnetic resonance; CSD, cardiac sympathetic denervation; CS, cardiac sarcoidosis; CD, clusters of differentiation; US, United States; VT, ventricular tachycardia; VF, ventricular fibrillation; ECG, electrocardiogram; EF, ejection fraction; EMB, endomyocardial biopsy; FDG, fluoro-deoxyglucose; GDMT, guideline-directed medical therapy; HRS, Heart Rhythm Society; HF, heart failure; ICD, implantable cardiac defibrillator; IVCD, intra-ventricular conduction delay; JCS, Japanese Circulation Society; LVAD, left ventricular assist device; LV, left ventricle; LGE, late-gadolinium enhancement; MRI, magnetic resonance imaging; NICM, non-ischemic cardiomyopathy; PET-CT, positron emission tomography–computed tomography; RV, right ventricle; ROC, receiver operating characteristic; SCD, sudden cardiac death; TNF, tumor necrosis factor.
